# A109 A SIMPLE METHOD TO CALCULATE ADENOMA DETECTION RATES

**DOI:** 10.1093/jcag/gwae059.109

**Published:** 2025-02-10

**Authors:** D C Sadowski, A Min, P Byun, T Miller

**Affiliations:** University of Alberta, Edmonton, AB, Canada; Alberta Health Services, Edmonton, AB, Canada; Alberta Health Services, Edmonton, AB, Canada; Alberta Health Services, Edmonton, AB, Canada

## Abstract

**Background:**

The adenoma detection rate (ADR) is a key indicator of the effectiveness of colonoscopy, which remains the gold standard for colorectal cancer detection. Many centers in Canada are increasingly adopting EPIC as their electronic health record system. However, the ADR calculation tool in the EPIC foundation build is not well-suited for Canadian practices, as it fails to account for colonoscopies performed due to positive fecal immunochemical tests (FIT) and relies on synoptic reporting of polyp histology by pathologists. To address this, we developed a straightforward text-based search strategy to identify adenomas in dictated pathology reports.

**Aims:**

This study aims to compare the accuracy of our new ADR metric against calculations based on manual reviews of pathology reports.

**Methods:**

We analyzed all screening colonoscopies conducted in Alberta from January to August 2024. For cases with specimens submitted for pathology, we examined the reports using a text string search for the terms “adenoma,” “tubular adenoma,” and “tubulovillous or villous adenoma” in the Final Diagnosis section. Reports were excluded if they contained the phrases “no adenoma,” “history of adenoma,” or “serrated adenoma.” Our reference standard was a manual review of pathology reports performed by trained polyp reconciliation nurses.

**Results:**

From January to August 2024, a total of 46,364 screening colonoscopies were conducted, with 45% of the patients being female. Manual record reviews revealed that 56% of cases had at least one adenoma, 20% had at least one sessile serrated lesion, 171 cases included at least one traditional serrated adenoma, and 345 cases of colorectal cancer were detected. Using manual chart review as the reference standard, our text-based ADR metric demonstrated a sensitivity of 81% and a specificity of 70%.

**Conclusions:**

The text search-based ADR metric shows promise in simplifying the calculation of ADR, especially for centers that lack synoptic pathology reporting or the capacity for manual pathology report reviews. Further research is necessary to enhance the accuracy of this metric.

**Funding Agencies:**

None

